# The Need for Standardized Guidelines for the Use of Monocyte Distribution Width (MDW) in the Early Diagnosis of Sepsis

**DOI:** 10.3390/jpm15010005

**Published:** 2024-12-27

**Authors:** Andrea Piccioni, Fabio Spagnuolo, Silvia Baroni, Gabriele Savioli, Federico Valletta, Maria Chiara Bungaro, Gianluca Tullo, Marcello Candelli, Antonio Gasbarrini, Francesco Franceschi

**Affiliations:** 1 Department of Emergency Medicine, Fondazione Policlinico Universitario A. Gemelli-IRCCS, 00168 Rome, Italy; andrea.piccioni@policlinicogemelli.it (A.P.); federico.valletta@policlinicogemelli.it (F.V.); mariachiara.bungaro@guest.policlinicogemelli.it (M.C.B.); gianluca.tullo@guest.policlinicogemelli.it (G.T.); marcello.candelli@policlinicogemelli.it (M.C.); francesco.franceschi@policlinicogemelli.it (F.F.); 2 Facutly of Medicine and Surgery, Università Cattolica del Sacro Cuore, 00168 Rome, Italy; silvia.baroni@unicatt.it (S.B.); antonio.gasbarrini@policlinicogemelli.it (A.G.);; 3 Unit of Chemistry, Biochemistry and Clinical Molecular Biology, Department of Laboratory and Hematological Sciences, Fondazione Policlinico Universitario Agostino Gemelli-IRCCS, Università Cattolica del Sacro Cuore, 00168 Rome, Italy; 4 Department of Emergency Medicine and Surgery, IRCCS Fondanzione Policlinico San Matteo, 27100 Pavia, Italy; g.savioli@smatteo.pv.it; 5 Medical and Surgical Science Department, Fondazione Policlinico Universitario A. Gemelli-IRCCS, Università Cattolica del Sacro Cuore, 00168 Rome, Italy

**Keywords:** MDW, sepsis, monocytes, PCT, PCR, VCS, CBC, WBC

## Abstract

Sepsis is a complex and potentially life-threatening syndrome characterized by an abnormal immune response to an infection, which can lead to organ dysfunction, septic shock, and death. Early diagnosis is crucial to improving prognosis and reducing hospital management costs. This narrative review aims to summarize and evaluate the current literature on the role of monocyte distribution width (MDW) as a diagnostic biomarker for sepsis, highlighting its advantages, limitations, and potential clinical applications. MDW measures the volumetric distribution width of monocytes, reflecting monocytic anisocytosis, and is detected using advanced hematological analyzers. In 2019, it was approved by the FDA as a biomarker for sepsis due to its ability to identify systemic inflammatory response at an early stage. Thirty-one studies analyzed by us have shown that an increased MDW value is associated with a higher risk of sepsis and that its combination with clinical parameters (such as qSOFA) and other biomarkers (CRP, PCT) can enhance diagnostic sensitivity and risk stratification capacity. Despite its high sensitivity, MDW has lower specificity compared to more established biomarkers such as procalcitonin, thus requiring a multimodal integration for an accurate diagnosis. The use of MDW in emergency and intensive care settings represents an opportunity to improve early sepsis diagnosis and critical patient management, particularly when combined with other markers and clinical tools. However, further studies are needed to define a universal cut-off and confirm its validity in different clinical contexts and pathological scenarios.

## 1. Introduction

### 1.1. Presentation for MDW

Monocyte distribution width (MDW) is a biomarker that measures the variation in monocyte volume, reflecting monocytic anisocytosis. It is assessed using state-of-the-art hematological analyzers such as the UniCel DxH800/900 (Beckman Coulter, Miami, FL, USA), which are quantitative, multiparametric, and fully automated systems [1]. Monocytes are identified based on their individual cell volume, high-frequency conductivity, and laser light scatter. MDW, calculated using VCS technology, enables the assessment of the volumetric distribution of these cells [2]. In 2019, MDW was approved by the U.S. Food and Drug Administration (FDA) as a diagnostic biomarker for sepsis.

### 1.2. General Characteristics of Sepsis

Sepsis is a complex and potentially life-threatening syndrome caused by a dysregulated host response to infection, which leads to organ dysfunction and can progress to septic shock and death [3]. Septic shock, in turn, should be defined as a subset of sepsis characterized by significant cellular, metabolic, and circulatory abnormalities, which are associated with higher mortality compared to sepsis alone. The most common pathogens responsible for sepsis include bacteria such as Escherichia coli, Klebsiella pneumoniae, and Staphylococcus aureus [4]; however, viruses (e.g., SARS-CoV-2), fungi, and parasites may also be involved. Sepsis is one of the leading causes of preventable in-hospital deaths, and its early management can significantly reduce mortality and decrease hospital management costs. A study by Paoli et al. demonstrated a reduction of approximately 1.5 days in the length of hospital stay through early diagnosis using MDW, leading to improved outcomes and reduced hospital costs [5].

The definitions of sepsis have undergone a revision with the introduction of Sepsis-3 in 2016. While Sepsis-2 (2001) was based on the clinical criteria of systemic inflammatory response syndrome (SIRS), Sepsis-3 introduced a more dynamic concept centered on organ dysfunction, measured through the Sequential Organ Failure Assessment (SOFA) score [6]. The SOFA score assesses the respiratory, cardiovascular, hepatic, coagulation, renal, and neurological systems, with an increasing score indicating worsening organ function [3]. In emergency departments, a simplified version called quick-SOFA (qSOFA) is often used for practical purposes to quickly identify patients at risk of sepsis based on three parameters: systolic blood pressure, respiratory rate, and altered mental status [7].

In high-income countries, there are approximately 31.5 million cases of sepsis and 19.4 million cases of severe sepsis annually worldwide, resulting in approximately 5.3 million deaths each year, with a mortality rate ranging from 15% to 50% [8]. Early diagnosis is essential, as it allows for timely treatment with antibiotics, fluids, and hemodynamic support. However, diagnosis remains challenging due to the variability in clinical manifestations and the lack of a single specific marker [9]. About 258 biomarkers have been studied in sepsis, most of which have been evaluated in fewer than five studies [10], but none have achieved the necessary sensitivity and specificity to be adopted alone in clinical practice.

### 1.3. Role of Immunity in Sepsis

Sepsis is characterized by two main phases: the hyperimmune phase and the immunosuppressive phase, which are initiated by the action of various immune cells that trigger a series of immune responses and by the interactions among these cells [11].

#### 1.3.1. Neutrophils

At the onset of infection, the innate immune system activates immediately, with neutrophils being the first phagocytes to migrate from the blood to the site of infection [12]. There, they release reactive oxygen species (ROS), inflammatory mediators, and antimicrobial proteins, forming neutrophil extracellular traps (NETs). These are reticular structures made of DNA that trap and neutralize pathogens [13,14]. In the early stages of sepsis, NETs serve as a physical barrier against pathogens; however, as the disease progresses, NETs can exacerbate tissue damage by promoting inflammation and facilitating thrombus formation, acting as damage-associated molecular patterns (DAMPs) [15].

In sepsis, neutrophils rapidly increase in the blood due to their prolonged survival and inhibition of apoptosis [16,17]. This surge utilizes the reserves of the bone marrow, which releases immature neutrophils that are less capable of recognizing and phagocytizing pathogens [18]. The presence of these immature neutrophils hinders the activation of other immune cells, such as lymphocytes, thereby predisposing the host to the development of immunosuppression in advanced sepsis [19,20]. Additionally, these immature neutrophils exhibit reduced deformability, which facilitates their accumulation in capillaries, leading to vascular occlusion, tissue hypoxia, and organ damage [21]. This is further linked to the enhanced interaction between ICAM-1 (on endothelial cells) and β2 integrins (on neutrophils), promoting their adhesion and vascular infiltration [22]. In the advanced phase of sepsis, neutrophils display abnormal migration and functionality, failing to effectively reach the infected sites and instead accumulating in critical organs. This results in significant damage as they infiltrate organs such as the liver and kidneys, releasing additional inflammatory mediators that worsen tissue injury and accelerate the progression of sepsis [23].

Cytokines such as TNF-α, IL-1β, and IL-6, along with bacterial components, activate the growth factor G-CSF, stimulating neutrophil production [24]. Once activated, neutrophils generate a significant amount of reactive oxygen species (ROS) during the respiratory burst, which damages tissues through the release of bactericidal substances and leads to mitochondrial injury [25]. Additionally, the excessive ROS levels compromise vascular endothelial cells, increasing vascular permeability and contributing to systemic dysfunction [26,27].

#### 1.3.2. Monocytes and Macrophage

**Figure 1:**
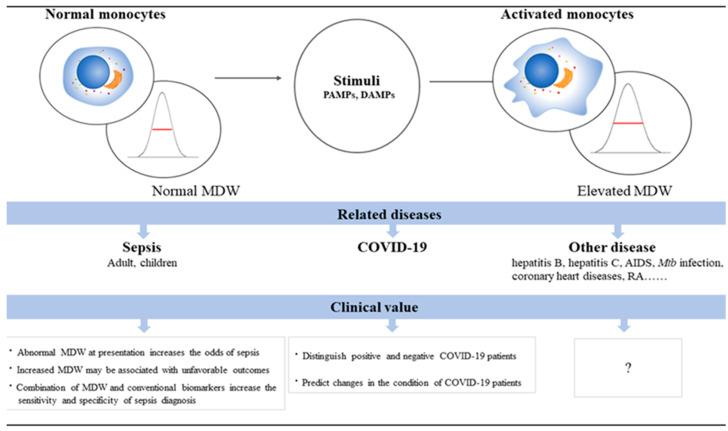
Morphological changes in monocytes associated with altered MDW levels [52].

In sepsis, monocytes undergo significant morphological and functional changes (Figure 1), and their increase corresponds to a strong inflammatory response. Innate immunity, activated by DAMPs and PAMPs, represents the first line of defense against infections [28], with monocytes playing a crucial role in the immune response [29,30,31]. Anisocytosis, defined as the variation in cell size, is a hallmark of abnormal monocyte activation [32]. Additionally, monocytes are classified into subgroups based on chemokine receptor expression, which leads to an increase in MDW levels [29]. In the septic response, monocytes become activated and differentiate into pro-inflammatory macrophages that release cytokines such as TNF-α, IL-1, and IL-6 [30]. These pro-inflammatory factors contribute to the systemic inflammation that characterizes sepsis.

Once activated, monocytes differentiate into macrophages, playing a significant role in modulating the inflammatory response. Under septic conditions, monocytes tend to produce fewer pro-inflammatory cytokines and enter a state of immune “paralysis”, a phenomenon known as endotoxin tolerance [33]. Monocytes play various roles in sepsis, such as pathogen elimination through phagocytosis, antigen presentation to activate other immune cells, and secretion of both pro-inflammatory and anti-inflammatory cytokines, thereby modulating the immune response.

Macrophages, as key cells of the innate immune system, are crucial in responding to sepsis due to their capacity to adapt and differentiate into subtypes with distinct functions. They can polarize towards the M1 phenotype, characterized by a pro-inflammatory response, or the M2 phenotype, which instead promotes the resolution of inflammation and tissue repair [34].

M1 macrophages are activated by pro-inflammatory signals, such as Th1 cytokines (e.g., TNF-α and IFN-γ) and pathogen-associated molecular patterns (PAMPs) like lipopolysaccharide (LPS) [35]. These macrophages exhibit high expression levels of markers such as CD68, CD80, CD86, and MHC-II, which are essential for antigen presentation and the activation of adaptive immunity [36]. Additionally, M1 macrophages produce significant amounts of nitric oxide (NO) via the enzyme iNOS and release pro-inflammatory cytokines (e.g., IL-6, IL-12, IL-23, IL-1β) along with reactive oxygen and nitrogen species [37]. Prolonged M1 polarization can lead to tissue damage and multi-organ dysfunction due to sustained inflammation.

In later stages of sepsis, the M2 macrophage phenotype becomes predominant. M2 macrophages are activated by anti-inflammatory signals, such as Th2 cytokines (IL-4, IL-13), TGF-β, and immune complexes [38]. This response supports inflammation resolution, tissue repair, and angiogenesis, though it can also lead to immune paralysis, increasing the risk of secondary infections and complications in septic patients [39].

#### 1.3.3. Lymphocytes

T and B lymphocytes are crucial for immunological memory and targeted immune responses. T lymphocytes are particularly central to adaptive immunity; upon activation, they proliferate and localize to infected tissues, directing the immune response [40]. TH1 cells, through cytokines like IFN-γ and TNF-α, enhance macrophage phagocytic and bactericidal functions, intensifying the inflammatory response necessary to combat infections effectively. Concurrently, CD8+ T cells target and eliminate infected cells, while regulatory T cells (CD4+, CD25+, also known as Tregs) moderate the immune response, preventing unnecessary damage to healthy tissues [41,42].

In sepsis, however, the excessive activation of T lymphocytes can become detrimental. The massive release of pro-inflammatory mediators, such as IFN-γ, exacerbates the inflammatory response, leading to significant tissue and organ damage. In response to this hyper-inflammatory state, the body triggers anti-inflammatory mechanisms, resulting in the production of mediators like TGF-β and IL-10 to balance the immune reaction [43]. Although essential, this counterbalance can induce an immunosuppressive state marked by reduced and functionally inhibited lymphocytes, often culminating in immune paralysis [39]. In this condition, the patient becomes highly susceptible to recurrent infections and prolonged organ dysfunction, as the compromised immune system struggles to effectively clear pathogens [44].

#### 1.3.4. Cell Interactions

Interactions between lymphocytes, neutrophils, and monocytes are crucial in the immune response to sepsis. In sepsis, innate and adaptive immune cells engage in a complex, yet essential, and sometimes destructive, role in defending the body against infection. Beyond their primary function of phagocytosing and removing pathogens, innate cells (such as macrophages and dendritic cells) generate pathogen-derived antigens, enabling the adaptive immune system to recognize and respond specifically to invaders [45]. In sepsis, the interaction among various immune cells is critical in understanding the progression of the inflammatory response.

Neutrophils, typically involved in the innate response, can negatively impact T lymphocytes through mechanisms such as expressing PD-L1 in response to IFN-γ, which inhibits T-cell activation and proliferation while promoting apoptosis [46]. This imbalance between T-helper cell subtypes (Th1 and Th2), particularly with an excess of Th2 signaling and IL-4, leads to an immunosuppressive state that impairs the body’s ability to respond effectively to infection. Sepsis often results in lymphopenia due to both decreased production and increased apoptosis of these cells, a phenomenon that contributes to the characteristic immunosuppression observed in the late stages of sepsis.

Lymphocytopenia does not directly influence MDW; however, the release of histones following apoptosis may contribute to MDW variations. In a study by *Ligi* et al. [47] MDW values significantly increased in a dose- and time-dependent manner following histone administration (highly conserved, intranuclear, positively charged proteins). These proteins can alter cytoplasmic granularity, cell volume, and nuclear structure in monocytes, thereby promoting heterogeneity without affecting cell count. After three hours of treatment, nearly all cytokines increased significantly in a dose-dependent fashion. The most prominent response was observed in significantly elevated G-CSF levels, along with increases in IL-1β, IL-6, MIP-1β, and IL-8. This study may reflect monocyte anisocytosis and cytokine storm seen in sepsis and COVID-19. Furthermore, neutrophilic leukocytosis and lymphopenia have been observed as predictors of progression to a more severe form of COVID-19, correlating with increased mortality and poor outcomes [48]. Pearson correlation analysis between MDW and blood test results for all patients included in the study showed that MDW was negatively correlated with total platelets (PLT) (r = −0.140, *p* < 0.001), lymphocyte percentage (LY%) (r = −0.168, *p* < 0.001), and monocyte percentage (MO%) (r = −0.262, *p* < 0.001). Kim et al., in a retrospective single-center study, confirmed that lymphocyte and platelet counts decreased with disease severity, while MDW increased [49].

MDW (monocyte distribution width) assesses the range of monocyte distribution, which can signal an inflammatory response, as sepsis is known to increase the morphological variability of monocytes. Although immunoparalysis in sepsis may lower cytokine production, morphological variability in monocytes often remains and can serve as a reliable activation indicator, particularly when considering timing. A simplified in vitro study demonstrated the key role of the inflammasome in transforming monocytes into swollen cells that undergo pyroptosis [50]. These findings link MDW to early events in the host’s dysregulated response to infection, supporting its potential to predict progression to sepsis in patients without evident clinical manifestations. In another study, an elevated MDW at presentation predicted progression to Sepsis-3 within 72 h in 71% of cases [51]. This parameter might be more dependable in the early stages of sepsis, while in advanced stages, its specificity in distinguishing other inflammatory or tissue-damage causes may diminish. Further studies are necessary to clarify and support MDW’s utility across different sepsis stages. This narrative review aims to summarize the current literature on the diagnostic performance of MDW (highlighting its advantages and disadvantages) and to provide guidance on its appropriate and informed use in clinical practice.

## 2. Materials and Methods

For this narrative review, we examined the scientific literature from the last fifteen years to identify studies and reports related to the diagnostic and prognostic accuracy of MDW in emergency departments (EDs) and intensive care units (ICU). The search was conducted on PubMed using specific keywords: “MDW”, “MDW and infection”, “MDW and sepsis”, “MDW and ED”, “MDW and ICU”, “MDW and PCT and PCR”. We narrowed the selection to articles in English, primarily focusing on observational studies, systematic reviews, and meta-analyses. Initially, 72 articles were identified, from which those related to the pediatric population were excluded, resulting in a total of 45 studies (Table 1), 96% of which were published in the last five years. We excluded the pediatric population because our study is aimed at an adult clinical care setting (patients > 18 years).

## 3. Results

### 3.1. MDW Applications in Emergency Departments

MDW has been adopted in many emergency departments (EDs). Due to its favorable characteristics, it could become an integral part of triage protocols for critically ill patients. The combined use of MDW with clinical scores, such as qSOFA, has shown improved early identification of septic patients [53], reducing diagnostic time and improving patient outcomes. However, in a study involving a sample of 2158 patients recruited across three different emergency departments, it was found that, independent of SIRS or qSOFA variables, an initial MDW > 20.0% was associated with a nearly six-fold increase in the likelihood of Sepsis-2 and a four-fold increase in the likelihood of Sepsis-3 [54]. In another emergency department cohort, at an MDW cut-off of 20.0, the sensitivity was 0.76 and the specificity 0.35 for Sepsis-3; at the same cut-off, the sensitivity for infection was 0.72 and specificity 0.64 [55].

Early identification of septic patients significantly improves when multiple tools are combined. Hou et al. demonstrated moderate sensitivity and specificity associated with MDW and a higher diagnostic capacity when considering a combination of qSOFA > 1, neutrophil-to-lymphocyte ratio (NLR) > 9U, platelet-to-lymphocyte ratio (PLR) > 210, and MDW > 20 [56]. Subsequent studies by Polilli et al. and Malinovska et al. further supported the integration of MDW into clinical decision making to enhance sepsis screening [57,58]. There is considerable evidence supporting MDW’s diagnostic validity. Crouser et al., in a prospective cohort study, demonstrated that an MDW value above 20.0 U, either alone or in combination with WBC, could diagnose sepsis early according to Sepsis-2 or Sepsis-3 criteria [51]. Another study recently identified the best thresholds to exclude infection (MDW ≤ 17) and sepsis (MDW ≤ 18) [59].

Agnello et al. [2] conducted a multicenter cohort study in three emergency departments, enrolling 2215 subjects divided into four groups: control group, infection group, SIRS group, and sepsis group. MDW levels were significantly higher in sepsis patients compared to all other groups (*p* < 0.001) [2]. A similar study by Poz et al. reported an MDW sensitivity of 87.3% and specificity of 71.7% at a cut-off of 20.1, indicating that MDW was effective for early sepsis recognition [60]. Supporting this, a study by Singla et al. conducted in India also highlighted its diagnostic utility [61]. In a retrospective, single-center cohort study of adult COVID-19 patients, MDW was shown to predict sepsis. The mean MDW was significantly higher in cases of sepsis (25.50 ± 5.93) compared to those without sepsis (23.13 ± 4.46) (*p* < 0.01), correlating with respiratory failure/hypoxia and death. An MDW value of 24.9 was identified as the optimal cut-off for determining fatal outcomes [62].

A previous prospective cohort study by Crouser et al., conducted in two different emergency departments and enrolling 1320 subjects, divided patients into the typical four groups: control, SIRS, infection, and sepsis. Parameters such as mean neutrophil volume (MNV), neutrophil distribution width (NDW), mean monocyte volume (MMV), and MDW were measured using the UniCel DxH 800 analyzer along with routine complete blood count (CBC). The results showed that MDW was an excellent discriminant of sepsis compared to other conditions (AUC, 0.79; 95% CI, 0.73–0.84) and was associated with sepsis severity, with higher values in patients with organ dysfunction. Additionally, MDW significantly enhanced the diagnostic performance when combined with WBC (AUC, 0.89 for MDW + WBC vs. 0.81 for WBC alone; *p* < 0.01), supporting its diagnostic potential alone or, even more effectively, in combination with WBC [63]. In another study, MDW and other parameters were significantly correlated with sepsis-induced acute kidney injury (AKI) [64].

Therefore, this laboratory parameter also provides prognostic information: elevated MDW levels are associated with a higher probability of multiple organ dysfunction and clinical deterioration. Values above 25 indicate a higher likelihood of severe sepsis, while a cut-off around 20, as previously described, can be used to identify at-risk patients. Its prognostic validity has been more thoroughly investigated in intensive care unit studies (Table 2).

**Table 2:** Summarization of the different analyzed studies in ED.

Author	Type	Year	Department	Subjects	Results	Cut-Off
Agnello et al. [2]	Observational study	2021	ED	2215 Classified according to Sepsis-2 criteria.	MDW is an optimal diagnostic marker for sepsis.	23.5
Crouser et al. [51]	Observational study	2019	ED	2212	MDW is an effective diagnostic marker for sepsis; in association with WBC, it improves diagnostic performance.	20
Woo et al. [53]	Observational study	2021	ED	549 Sepsis-3 criteria.	The sensitivity was 83.0% for MDW, 69.7% for CRP, and 76.6% for PCT. The combination of MDW and qSOFA improves the diagnostic performance.	24
Crouser et al. [54]	Observational study	2020	ED	2158 including 385 in Sepsis-2, 243 in Sepsis-3.	MDW is an effective diagnostic marker for sepsis.	20
Cusinato et al. [55]	Observational study	2023	ED	2570	MDW is an optimal diagnostic marker for sepsis.	20
Hou et al. [56]	Observational study	2021	ED	1480	MDW is an optimal diagnostic marker for sepsis; when combined with other biomarkers and scores, its diagnostic performance is superior.	20
Polilli et al. [57]	Observational study	2022	ED	2724	MDW is an optimal diagnostic marker for sepsis.	22
Malinovska et al. [58]	Observational study	2022	ED	7952	MDW is an effective diagnostic marker for sepsis.	24.8
Kralovcova et al. [59]	Observational study	2024	ED	1925	MDW has good diagnostic performance in the diagnosis of infection and sepsis.	22
Poz et al. [60]	Observational study	2022	ED	985	MDW is an effective diagnostic marker for sepsis; in association with WBC, it improves diagnostic performance.	20.1
Singla et al. [61]	Observational study	2022	ED	148	MDW is an optimal diagnostic marker for sepsis.	20
Frugoli et al. [62]	Observational study	2023	ED	331	MDW is an optimal diagnostic and prognostic marker for sepsis.	24.9
Crouser et al. [63]	Observational study	2017	ED	1320	The integration of MDW with the white blood cell count improves the detection of sepsis compared to the white blood cell count alone.	20.5
Pan et al. [64]	Observational study	2024	ED	19,792 Sepsis-2	MDW, RDW, and NLR, along with the initial SOFA score and MAP, can help identify acute kidney injury induced by sepsis.	20.8
Li et al. [65]	Observational study	2022	ED	402	MDW is a more sensitive biomarker than PCT in predicting SIRS and Sepsis-3.	
Hausfater et al. [66]	Observational study	2021	ED	1517 including 260 in Sepsis-2, 144 in Sepsis-3.	The diagnostic performance of MDW combined with WBC was similar to that of PCR alone but higher than that of PCT.	
Encabo et al. [67]	Observational study	2023	ED	102	MDW with this cut-off allows distinguishing between infected and non-infected patients.	20.115
Jo et al. [68]	Observational study	2022	ED	1404 in Sepsis-3	MDW is an optimal diagnostic marker for sepsis.	21.7
Agnello et al. [69]	Observational study	2021	ED	2215	The sepsis index is a good diagnostic parameter for sepsis.	

### 3.2. MDW Applications in Intensive Care Units

In the ICU setting, changes in MDW from day 1–4 and 5 have been significantly associated with mortality or survival [70]. Similar findings emerged in a previous study, where an MDW of 26.20 allowed discrimination between survivors and non-survivors with a sensitivity of 77.8% and specificity of 67.6% [71]. A similar cut-off was identified by Riva et al. Through ROC curve analysis, they determined an MDW value of 26.4 as the optimal threshold for assessing the probability of a fatal outcome during disease progression, resulting in a promising area under the curve (AUC) of 0.76 (95% CI: 0.66–0.87; sensitivity, 0.75; specificity, 0.70), with a negative predictive value (NPV) of 0.93. These findings indicate a strong prognostic value [72].

Agnello et al., in an ICU study, endorsed the use of MDW as a sepsis biomarker. MDW levels in sepsis patients were significantly higher than in those without sepsis or in patients who developed sepsis during hospitalization [73]. Patients who developed sepsis showed an increase in MDW values at the time of clinical diagnosis compared to admission levels [73,74]. In a study by Polilli et al., MDW values > 23 were associated with a high positive predictive value (PPV) for sepsis, while MDW values ≤ 20 were associated with a high negative predictive value (NPV), suggesting its potential to monitor ICU patients for sepsis development. The NPV for MDW > 20 reached 100%, as this category included all bacteremic patients [75].

The potential prognostic validity was further investigated in a study by Bajic et al. on 28-day mortality in COVID-19 sepsis. A significant association was found between MDW, lactate, procalcitonin, and fatal outcomes: higher values were reported in the deceased group [76]. Another study by Alsuwaidi et al. previously revealed that an MDW ≥ 24.685 is strongly correlated with an unfavorable prognosis in COVID-19 [48] (Table 3).

**Table 3:** Summarization of the different analyzed studies in ICU.

Author	Type	Year	Department	Subjects	Results	Cut-Off
Alsuwaidi et al. [48]	Observational study	2022	ICU	2454	MDW has a prognostic role.	24.7
Kim et al. [49]	Observational study	2022	ICU	87	MDW is a potential early predictor of COVID-19 severity and can be easily assessed through a CBC.	
Agnello et al. [70]	Observational study	2024	ICU	193 Sepsis-3 criteria.	MDW is an optimal diagnostic and prognostic marker for sepsis.	
Liu et al. [71]	Observational study	2023	ICU	252	MDW is an optimal diagnostic marker for sepsis.	
Riva et al. [72]	Observational study	2021	ICU	87	MDW has a prognostic role.	26.4
Agnello et al. [73]	Observational study	2020	ICU	96	MDW is an optimal prognostic marker for sepsis.	21.4
Polilli et al. [75]	Observational study	2021	ICU	211	MDW is an optimal diagnostic marker for sepsis.	23
Baijic et al. [76]	Observational study	2023	ICU	160	MDW has a prognostic role.	
Piva et al. [77]	Observational study	2021	ICU	506	MDW increase is not affected by the aetiology of sepsis.	24.63
Motawea et al. [78]	Meta-Analysis	2023	ICU and ED	1348	The diagnostic accuracy of MDW is higher than that of PCT.	
Huang et al. [79]	Meta-Analysis	2023	ICU and ED	22,459	MDW is an effective diagnostic marker for sepsis.	
Malinovska et al. [80]	Meta- Analysis	2023	ICU and ED	34,124	MDW is an effective diagnostic marker for sepsis.	
Agnello et al. [81]	Meta-Analysis	2022	ICU and ED	9475	MDW represents a reliable biomarker for sepsis screening.	
Meraj et al. [82]	Observational study	2023	ICU	111	MDW represents a reliable biomarker for sepsis screening; MDW has predictive capabilities similar to PCT and CRP.	20.24

### 3.3. MDW Compared to CRP and PCT

In the blood sample analyzed by Agnello et al., levels of CRP, white blood cells (WBC), and MDW were determined, revealing a statistical correlation between MDW and CRP. The area under the curve (AUC) was significantly higher for MDW compared to CRP, indicating better diagnostic accuracy for MDW [2]. MDW demonstrated similar diagnostic performance to CRP and PCT. Specifically, MDW is more sensitive than PCT [65] in the early diagnosis of sepsis but less specific. PCT, on the other hand, has the advantage of better distinguishing between bacterial and viral infections—a discriminating power absent in MDW. Lastly, CRP is slower to respond to inflammatory stimuli.

In a multicenter clinical study [66] conducted in two large European emergency departments, an MDW cut-off of 21.5 in K3 EDTA tubes showed a sensitivity of 75% [69–80, 95% CI] and a specificity of 73% [70–75, 95% CI], making it a good screening tool. The AUC of MDW combined with WBC was similar to CRP alone (0.85 [0.83–0.87]) and surpassed that of PCT in diagnosing sepsis. The combination of biomarkers did not improve the AUC further. Another study with the classic three patient groups—non-infection, infection, and sepsis—showed that the sensitivity was 83.0% for MDW (cut-off, 19.8), 69.7% for CRP (cut-off, 4.0), and 76.6% for PCT (cut-off, 0.05). The combination with qSOFA appeared to enhance the diagnostic performance of MDW alone [53].

In another observational study in an ICU by Piva et al., the diagnostic performances of PCT, CRP, and MDW were compared in predicting sepsis, concluding that MDW was comparable to PCT while outperforming CRP and WBC [77]. A meta-analysis of five studies indicated that the diagnostic accuracy of MDW was superior to that of PCT [78], while another meta-analysis equated the diagnostic performance of MDW to that of PCT and CRP, using data from eight and five studies, respectively [79]. Although the results are encouraging [80,81], the original studies reported in the recent meta-analyses were conducted in diverse clinical settings, using inconsistent cut-off values for MDW and variable outcome definitions.

Therefore, while MDW shows promise as an early sepsis marker, further standardization of study protocols and cut-off values is necessary to confirm its utility across different clinical environments and patient populations (Table 2 and Table 3).

### 3.4. Cut-Off Values

Various studies conducted to date show discrepancies in the optimal cut-off value for MDW as a sepsis predictor, with values varying depending on the clinical setting. The reported thresholds range between 19 and 25, with higher cut-offs observed in ICU patients compared to those in the emergency department (ED) with early-stage sepsis. For example:

■ Piva et al. identified an MDW threshold of >24.6 in ICU patients [77].
■ Crouser et al. established a cut-off of >20 for early detection in the ED [63].
■ Agnello et al. reported a threshold of >23.5 [2].
■ Woo et al. suggested a cut-off of >19.8 [53].
■ Polilli et al. proposed a threshold of >21.9 [75].
■ Meraj et al. indicated a significant value at >20.24 [82].

In a smaller sample of 102 patients, an MDW cut-off of 20.115 provided the best diagnostic accuracy for identifying infected patients, with a sensitivity and specificity of 89.2% [67]. This variability in cut-offs may be due to differences in patient populations and the clinical settings in which the studies were conducted (e.g., ICU vs. ED). Another possible factor is the type of anticoagulant used in the sample (K3-EDTA vs. K2-EDTA). In a study involving 274 samples from healthy donors using EDTA-K2, the reference interval (RI) estimated using a non-parametric method was 14.77–21.13 [83]. Another study identified an RI between 16.2 and 23.1 [84] (Table 2).

These inconsistencies highlight the need for further research to establish a reliable and universally accepted MDW cut-off point that can be applied in diverse clinical contexts, ensuring the accuracy and consistency of this biomarker in sepsis screening and diagnosis.

## 4. Discussion and Conclusions

MDW (monocyte distribution width) is emerging as a crucial biomarker in the early diagnosis of sepsis, which increasingly requires timely intervention to improve clinical outcomes. Numerous studies have demonstrated the diagnostic efficacy of MDW in sepsis. Advantages and limitations have been identified in this regard. Thus, the main advantages of MDW are its high sensitivity and ease of measurement:

- MDW is available within the CBC, making it easily measurable and cost-effective;
- Its strength lies in its sensitivity, which reduces the occurrence of false negatives.

Therefore, its good negative predictive value (influenced by sensitivity) makes it a useful tool for ruling out sepsis in low-risk patients. Another positive feature is that no correlations with demographic factors such as age, sex, or surgical conditions have been observed. No significant differences were noted in relation to the pathogen type, with the exception of two studies analyzed [68,77]. In the first case, the aetiologic agent of sepsis was identified for each patient, and it was observed that the diagnostic performance of MDW was maintained regardless of whether the infection was viral, bacterial, or fungal. In the second study, elevated MDW values were also confirmed in both the group with positive blood cultures and the sepsis group caused by viral infections.

However, unlike bacterial sepsis, fungal sepsis is more frequently associated with leukopenia, involving a significant reduction in leukocytes that renders the immunocompromised patient more vulnerable to this type of infection [85]. Furthermore, in fungal infections, the capacity of macrophages and neutrophils to respond effectively to pathogens is compromised, leading to reduced phagocytic activity and an increased susceptibility to opportunistic infections [86].

The varying factor based on the different aetiological agents may be the monocyte subpopulation (considering CD14 and CD16 differentiation clusters). These include classical monocytes, intermediate monocytes, non-classical monocytes, and double-negative cells.

In a study by Cusinato et al. [87], sepsis samples showed a relative decrease in classical monocytes, accompanied by increases in the proportions of both intermediate cells (median 12.0%) and non-classical cells (median 9.3%). In contrast, COVID-19 samples revealed an apparent increase in the double-negative population (median 16.0%). This shift consistently resulted in an increase in MDW, without distinguishing this phenotypic difference.

Additional targeted research is needed to confirm or refute whether MDW retains its diagnostic accuracy regardless of the type of infection. Nevertheless, MDW remains a valuable marker for the early identification of sepsis and for monitoring residual monocyte function. This suggests that MDW may be suitable for screening any severe infection, so it could be an advantage but also a limitation because of its inability to distinguish bacterial infections, making it unsuitable for monitoring the response to antibiotic therapy over time.

While MDW offers valuable information in diagnosing sepsis, it has some limitations, such as lower specificity compared to other biomarkers like PCT (procalcitonin), which can result in false positives. Its diagnostic and prognostic performance is comparable to, or in some cases slightly better than, PCR and PCT; however, the limitation in specificity remains. For this reason, it is recommended to combine MDW with other parameters, such as the neutrophil/lymphocyte ratio and more established markers like PCT and CRP (C-reactive protein), to improve diagnostic accuracy. Compared to the sepsis index (SI, cut-off >1) derived from the MDW and mean monocyte volume (MMV), the SI showed higher specificity (94.7% vs. 90.6%), without any decrease in sensitivity (92.0%); additionally, the positive predictive value (PPV) was higher for the sepsis index [69]. Recent research has highlighted that when used in combination with clinical scores such as qSOFA, MDW can enhance the ability to identify septic patients early, reducing the time to diagnosis and improving outcomes [53]. Therefore, the combined use of MDW with other parameters and clinical scores can enhance diagnostic accuracy.

Agnello et al. analyzed the kinetic profile of MDW compared to other biomarkers. Exclusion criteria included pediatric patients, incomplete data collection, and underlying conditions, potentially associated with immune dysregulation, such as AIDS, organ or bone marrow transplants, and hematologic diseases, as well as hospital stays shorter than 5 days. Patients were assessed at several time points: at admission (T0), after 24 h (T24), 48 h (T48), and 72 h (T72). MDW showed statistically significant differences only at T0 and T24, with a declining trend towards T72. At 24 h, MDW continued to show a significant association with mortality, unlike the SOFA score, suggesting that MDW may be an independent predictor of mortality, beyond the initial SOFA score [88]. Lorubbio et al. used a ΔMDW calculated based on the difference between the first and third MDW measurements, taken approximately 5–7 days after hospitalization, in both surviving and non-surviving patients. In non-surviving patients, the mean MDW at the first point and subsequent points did not differ significantly, while in survivors, ΔMDW increased significantly from the third measurement. A ΔMDW value < 1 was indicative of an unfavorable prognosis [89].

Further studies are warranted to explore the speed and accuracy with which this biomarker can reflect changes in the body’s response to infection. In sepsis, the body activates both infection resistance mechanisms (i.e., the immune system’s capacity to combat pathogens) and disease tolerance mechanisms (i.e., the ability to limit tissue damage from inflammation). A limitation of MDW may be its inability to promptly capture these dynamic responses, potentially making accurate clinical status identification challenging.

Another limitation is the lack of a standardized cut-off for the diagnosis of sepsis. An MDW value greater than 20.0 U is associated with an increased likelihood of Sepsis-2 and Sepsis-3. Furthermore, MDW has been shown to provide prognostic information, as elevated MDW levels are correlated with a higher likelihood of multiple organ dysfunction and clinical deterioration. Values above 25 are associated with a greater probability of severe sepsis, while a cut-off of around 20 is useful for identifying at-risk patients. Research has also shown that the optimal cut-off for MDW may vary depending on the clinical context. We have reported studies conducted in different clinical settings (ED and ICU) and on samples treated with different anticoagulants (K2EDTA and K3EDTA).

It was also noted that patients without infection but with comorbidities had higher MDW levels. A study by Kralovcova et al. found higher MDW values in the non-infectious group among patients with diabetes mellitus and multimorbidity (defined as at least two chronic conditions). Notably, 50% of the comorbidity group had active cancer [59]. In another study, patients with underlying chronic diseases were excluded because comorbidities can contribute to organ failure [82]. Another study found no association with the Charlson Comorbidity Index (CCI) [75]. Additionally, in several studies, comorbidities were not recorded accurately [56]. Although most studies do not explicitly address associations with chronic diseases, given that sepsis is more common in patients with preexisting conditions [53,90], variations in cut-off values could also be influenced by this factor.

This variability suggests the importance of establishing clear and standardized guidelines for the use of MDW in clinical practice to ensure its effective use in different healthcare settings.

In conclusion, based on the studies reviewed, monocyte distribution width (MDW) cannot be incorporated into precision medicine due to its low specificity, which limits its ability to distinguish between inflammatory and infectious conditions. Furthermore, clinical evidence remains limited, and interindividual variability cannot be excluded.

Nevertheless, the implementation of MDW in triage protocols and patient monitoring in emergency departments and intensive care units represents a significant step forward in the diagnosis and management of sepsis. The ease of measurement and low cost of MDW make it a readily accessible biomarker. Further studies would also be necessary to validate its prognostic role and to better define its use in combination with other biomarkers like PCT and CRP, as well as to monitor the response to antibiotic treatment and to further investigate whether the diagnostic performance is maintained regardless of whether the infection is caused by bacteria, viruses, or fungi.

## Figures and Tables

**Table 1:** Type and number of articles identified.

Type	Number
Review	1
Observational Study	26
Meta-analysis	4

## Data Availability

The articles cited in this paper are available on PubMed^®^, UpToDate^®^ and Cochrane^®^.
